# Relative Value of Quinine in Large Doses, as a Remedy in Typhus

**Published:** 1843-05

**Authors:** 


*Relative value of Quinine in large doses, as a remedy in Typhus.*—The successful results of the practice of Sig. Broqua have been already noticed in the Lancet. But a commission appointed to examine into the correctness of a memoir addressed by Broqua to the French Academy of Medicine, reports that some of the cases cited in the memoir are not proved to have been of a veritably typhoid character, and in others no proof is adduced of the quinine administered having been the means of cure. The report of the commission (if not belied by the journal which informs us of its presentation) sarcastically enough remarks, that "one interesting fact confirmed by Sig. Broqua's memoir is the *harmlessness* (l'innocuite presque constante) *of the sulphate of quinine* in large doses;" and it recommends that the memoir should be *honorably* shelved! In the discussion that followed its reading, Piorry stated, that in typhus fever, with engorgement of the spleen, he had seen quinine prove serviceable, which had not been the case when the fever was unaccompanied with splenic lesion. Martin Solon, who had employed the remedy under the personal inspection of Sig. Broqua at the Hôpital Beaujon, admitted that in cases in which the fever assumed a remittant type quinine was useful, but that remittent typhus was rare—at least at Paris. "Of five severe cases of typhus fever, in which quinine had been given, death had resulted in three instances; and in the two others, recovery had only taken place after a considerable lapse of time, and without any evidence to show that the sulphate of quinine had been the means of hastening it. In the post-mortem examinations of the subjects who had died (says Martin Solon), I failed to detect any peculiar alteration that I could fairly attribute to the large doses of the sulphate; it had passed in a manner imperceptibly through the stomach and intestines. A symptom I observed in those who recovered from the disease was a remarkable depression of the circulation. In short, I consider the advantage attributed to the sulphate to be more than doubtful." Much doubt was afterwards expressed by several members of the academy as to the *innocuity* of large doses of quinia or its sulphate; but finally the terms of the report were adopted, and the memoir was shelved by a majority of voices.—*Gaz. des Hop.*—*Lond. Lancet.*

---

*Amount of Respiration relatively to Sex and Age.*—Bourgery, in a memoir read before the French Academy of Sciences, asserts that, *cæteris paribus*, respiration is vigorous in proportion as the individual is younger and thinner. No condition is so productive of energetic respiration as youth. The respiration in a man of a given age is double the amount of that of a woman of the same age. At thirty years of age, the period of plenitude of the respiratory powers in both sexs, a man usually respires (*respiration forcee*) from half a gallon to a gallon (2.5 to 4.3 litres) of air (per minute); and a woman, from a quart to less than half a gallon in the same time. A boy of fifteen respires nearly half a gallon; and an old man of eighty, about three pints of air (1.35 litre). A strong man of thirty respires as much as two men of feeble constitution, boys of fifteen, or strong women; and four women of feeble constitution, boys of seven, or old men of eighty-five years of age. The respiration of a strong woman is accordingly equal to half the above amount, as estimated by Bourgery. According to the same authority, the faculty of respiration becomes impaired throughout life by successive ruptures of the air-cells, which inevitably attend all extensive respiratory efforts and other causes. Though these become more frequent as age advances, they occur likewise in infancy, and from the mere respiratory act. All diseases of the lung, however slight, tend also to incapacitate that organ.

*Gaz. des Hop.—Lond. Lancet.*

